# Functional microRNA screening using a comprehensive lentiviral human microRNA expression library

**DOI:** 10.1186/1471-2164-12-546

**Published:** 2011-11-03

**Authors:** Jos B Poell, Rick J van Haastert, Francesco Cerisoli, Anne S Bolijn, Lisette M Timmer, Begoña Diosdado-Calvo, Gerrit A Meijer, Andre AFL van Puijenbroek, Eugene Berezikov, Roel QJ Schaapveld, Edwin Cuppen

**Affiliations:** 1 Hubrecht Institute, Cancer Genomics Center, and University Medical Center Utrecht. Uppsalalaan 8, 3584 CT Utrecht, The Netherlands; 2 InteRNA Technologies BV, Padualaan 8, 3585 CH Utrecht, The Netherlands; 3 Department of Pathology, VU University Medical Center, Amsterdam, The Netherlands

## Abstract

**Background:**

MicroRNAs (miRNAs) are a class of small regulatory RNAs that target sequences in messenger RNAs (mRNAs) to inhibit their protein output. Dissecting the complexities of miRNA function continues to prove challenging as miRNAs are predicted to have thousands of targets, and mRNAs can be targeted by dozens of miRNAs.

**Results:**

To systematically address biological function of miRNAs, we constructed and validated a lentiviral miRNA expression library containing 660 currently annotated and 422 candidate human miRNA precursors. The miRNAs are expressed from their native genomic backbone, ensuring physiological processing. The arrayed layout of the library renders it ideal for high-throughput screens, but also allows pooled screening and hit picking. We demonstrate its functionality in both short- and long-term assays, and are able to corroborate previously described results of well-studied miRNAs.

**Conclusions:**

With the miRNA expression library we provide a versatile tool for the systematic elucidation of miRNA function.

## Background

MicroRNAs (miRNAs) were discovered as a class of small regulatory molecules ten years ago [1-3]. These ~21 nucleotide (nt), small RNAs recognize partially complementary sequences on target mRNAs [4,5]. Following the initial discovery of miRNAs, substantial effort has gone into characterization of the canonical miRNA pathway [6,7] and into miRNA discovery; by identifying miRNAs in more species and by adding to the list of known miRNAs [8]. Although cDNA cloning and northern blotting techniques can be used to detect the most abundant miRNAs, the advent of massively parallel sequencing technologies has propelled the miRNA field, allowing for both discovery and quantification of all miRNAs in a given sample [9,10].

With the bulk of the miRNAs revealed in commonly studied species, the next challenge lies in elucidating the biological processes in which miRNAs play a role. Current bioinformatic approaches rely on the identification of partially complementary sequences in mRNAs to predict miRNA targeting. Yet these approaches still come with one limitation; the exact parameters governing targeting remain unknown. Several prediction algorithms have been developed to overcome this difficulty by ascribing different weights to key parameters, such as binding energy between target and miRNA, conservation of the target site, quality of the "seed pairing", et cetera [11]. Still no single algorithm emerges as the best performer [12], and most algorithms predict thousands of targets for each miRNA [13]. Combining different target prediction algorithms generates shorter list of targets by creating more stringent cut-offs. This can provide some enrichment in true positives, but at the cost of more false negatives [14]. In addition to bioinformatics prediction, several approaches to genome-wide experimental miRNA target identification have been developed. These experiments utilize Argonaute pull-down assays (HITS-CLIP and PAR-CLIP) [15,16], changes in mRNA levels [17], and protein expression after introduction or ablation of a specific miRNA [18-20]. These studies support that miRNAs indeed function by targeting hundreds of genes. Still, it is a daunting task to derive a function for a miRNA from these long lists of potential target genes. Despite progress in systematic approaches to find sets of related gene that are enriched within these long target lists [21], we are still far from satisfactory *in silico *prediction of miRNA function.

Alternatively, differential expression of a miRNA is commonly used to infer its function [22,23]. Identification of conditions where a specific miRNA is expressed versus an opposing condition where it is not, offers some clues as to the potential action of the miRNA. While this approach has been very successful in leading investigators to uncover miRNA functions, it still requires direct experimentation to prove effects due to miRNA activity beyond providing an only coincidental biomarker.

Another approach to determine the function of a miRNA is by knocking it down [24-26], or knocking it out, of the genome of a model organism [27-29]. Experimental knockdown of miRNAs may confirm or invalidate predicted functions, but it requires prior knowledge where a miRNA is expressed. Even with this knowledge, sufficient knockdown to demonstrate an observable effect is not guaranteed. Complete knockout delivers a clean result, but may not result in an obvious phenotype. Adding to this challenge is the possibility that many miRNAs may elicit only subtle changes or are redundant with other family members entirely. Indeed, only a fraction of all *C. elegans *miRNA families display pronounced abnormal phenotypes when deleted [30]. Given these challenges, knocking out a miRNA in mice or in a human cell line may often prove a fruitless endeavor.

In order to unravel the functions of specific miRNAs, while overcoming much of the challenges discussed above, we proposed to introduce or overexpress miRNAs in a system of interest. Moreover, we argue that it is more efficient to examine the effect of any miRNA for a predetermined phenotype, rather than blindly investigating one miRNA at a time. Such screens have been performed on different scales. Most are based on transfection of miRNA mimics [31]; synthetic RNAs that usually have a modified backbone. Although this approach ensures the presence of the mature miRNA in the target cells, a miRNA mimic is not processed via the canonical miRNA biogenesis pathway. This multi-cleavage process starts with the recognition of a hairpin in the primary transcript and ends with a mature miRNA produced from one or both of the arms of the hairpin [32]. Bypassing this physiological processing step has several implications. For instance, several variants of a miRNA from the same primary transcript can arise due to variations in the processing pathway, such as arm switching, non-templated additions of adenines or uracils, and variations in the 3' and 5' cleavage sites [33,34]. Besides the loss of miRNA variants, the most-widely used miRNA mimics have modified backbones to increase stability within the cell. Consequently, they are not cleared naturally from the cells as endogenous miRNAs. Furthermore, it is possible that miRNA mimic transfection achieves cellular concentrations beyond physiological relevance. On the other hand, loading into the RISC complex, which is essential for biological activity, is not necessarily efficient for mimic transfection, as this process is known to be coupled to hairpin processing [35,36]. To express transcripts for endogenous processing into mature miRNAs, plasmid [37] or viral vectors [38,39] can be employed. While the retroviral library described by Agami and colleagues is a valuable tool for miRNA functional screens in pooled format, the viral supernatants are not available as individual isolates amenable to arrayed high-throughput screens [38].

Here, we describe the construction and application of a lentiviral human miRNA expression library. This library contains 660 annotated human miRNAs and 422 candidate miRNAs [10,40]. All are expressed from their genomic backbone, ensuring physiological processing of the miRNAs. The library is organized for high-throughput screening to provide a resource for the systematic elucidation of miRNA function. In addition, all lentiviral miRNA expression constructs can be applied individually to evaluate primary results. Lastly, we demonstrate the utility of this library in various types of screens to present the miRNA expression library as a versatile tool to study miRNA function.

## Results and Discussion

### Approach and setup

We aimed for the construction of a miRNA expression library that fulfills four criteria: 1) the library contains all human miRNAs; 2) each miRNA is represented in a separate stock to allow for arrayed screening; 3) the library can be used over a wide range of different cell types; 4) the miRNAs are swiftly and stably overexpressed. The first two criteria will be discussed below. The third and fourth criteria were met by choosing a lentiviral expression system, employing the pCDH vector. The lentiviral particles express VSV-G, a glycoprotein that grants broad tropism [41]. The glycoprotein's receptor is a lipid component of the plasma membrane that occurs on most cell types over a wide range of species. Lentiviruses integrate into the host genome [42], but unlike other retroviruses, lentiviruses do not require cell division for genomic integration [43,44]. For instance, the lentivirus is able to transduce quiescent stem cells and terminally differentiated neurons [43]. By integrating into the host genome, the construct is retained through cell divisions and can be stably expressed for an indefinite period of time. We chose a lentiviral backbone with a puromycin-resistance cassette to be able to select for successfully transduced cells [45].

To create a comprehensive library containing all human miRNAs, we included all miRNAs known at the time we started composing the library. We obtained genomic loci from the widely adopted miRBase miRNA repository (version 14) [46]. We also included a set of candidate miRNAs from previous experiments [10,40]. Although we did not update the library after viral particles for the entire library were produced, some of the candidate miRNAs have since entered the registry. See additional file 1, table S1 for a list of the current annotation of all miRNAs in the library.

**Figure 1:**
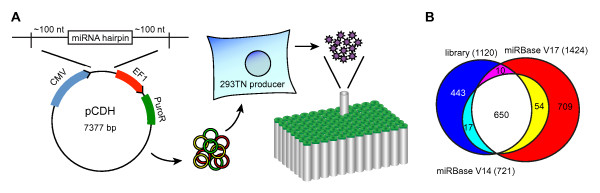
**Overview of the miRNA library**. (A) 660 annotated and 422 candidate miRNAs were cloned from their genomic loci into a lentiviral expression construct. From each construct, an individual batch of virus supernatant was created and portioned into individual tubes organized in a 96-well format. (B) Overlap between miRNAs included in the lentiviral library and miRNAs annotated in version 14 and version 17 of miRBase. The library contains constructs with variants or duplicates of some miRNA loci, which brings the total to 1120 constructs.

All miRNA loci, containing the full-length hairpin and ~100 nt flanking sequence on each side, were cloned into pCDH behind the CMV promoter. Care was taken to clone each miRNA separately, even when this miRNA resides in close proximity to another miRNA. After construction of the plasmid library, all constructs were packaged into lentiviral particles and portioned into individual tubes organized in ready-to-use 96-well format; the fact that tubes can be accessed separately adds versatility to the library. All virus supernatants were concentrated to titers of ~5*10^8 ^IFU/mL, in order to achieve efficient transduction with only small volumes of virus supernatant. This eliminates the need for infection under centrifugation or replacement of medium with virus-conditioned medium. While lentiviral particles were produced for all constructs individually, it should be mentioned the original plasmid library can also be applied in its own right. It can also be used for plasmid-based screens, recloning purposes, or to create a pool of constructs containing all or a subset of miRNA constructs for generating mixed lentiviral stocks. Figure 1 is a schematic of the library construction and its most important features.

### Library validation

**Figure 2:**
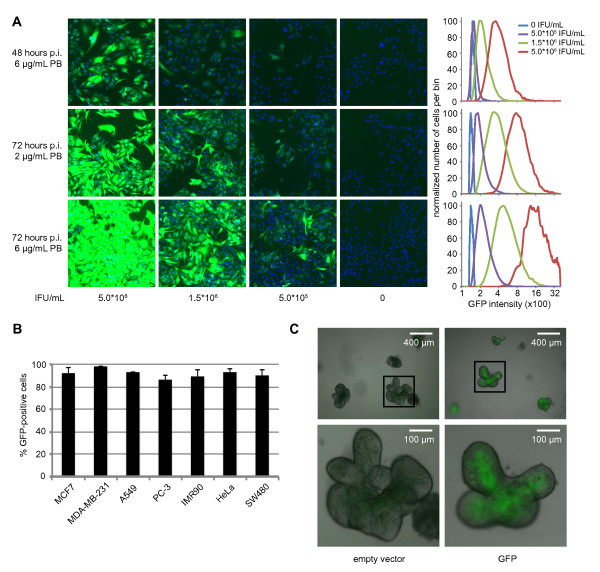
**Transduction efficiencies in different cell systems**. (A) A375 cells were infected with different amounts of GFP-encoding virus and different concentrations of polybrene. GFP intensity of each cell was measured after 48 hours and 72 hours. The graphs on the right summarize the GFP intensities of each population in the corresponding row. (B) Transduction efficiency of GFP-encoding virus for several cell lines. For all cell lines tested, transduction efficiencies above 80% were achieved. (C) Successful transduction of human intestinal stem cells with GFP-encoding virus. Infected stem cells were able to grow out into three-dimensional intestinal organoids in Matrigel. Below: magnification of inlay.

The first step in the characterization of the library is the determination of transduction efficiency and optimal titration of the lentiviral particles. To this end, we used a variant of pCDH containing a GFP cassette in place of the puromycin-resistance cassette. We infected A375 melanoma cells with different virus concentrations, in combination with several concentrations of polybrene. While transduction efficiency reached over 90% under several conditions, increased amounts of virus led to brighter GFP expression within the population of cells (Figure 2a). This indicates multiple copies are integrated per cell when using higher titers. GFP expression was higher at 72 hours after infection than after 48 hours. This implies 72 hours post infection is a more sensitive time point for determination of transduction efficiency, rather than earlier timepoints. We further optimized conditions for virus infection in a panel of seven cell lines (Figure 2b). Notably, this panel includes the diploid fetal lung fibroblast line IMR-90; a non-transformed cell line [47]. Optimal transduction efficiencies were reached between 10^6 ^and 10^7 ^IFU/mL.

As mentioned, lentivirus is a favorable vector for its ability to transduce cells that are hard to transfect, such as stem cells. We have successfully transduced primary human intestinal cells using a GFP-encoding vector. These cells were subsequently used to create intestinal organoids *in vitro *[48]. After prolonged culture, entire organoid bodies turned fluorescent, indicating the stem cells from which the organoids developed were also successfully transduced (Figure 2c).

While ectopic expression of GFP is useful to determine transduction efficiency, it does not address whether miRNAs are also successfully overexpressed. We used a panel of ten test miRNAs to assess miRNA overexpression in five human cell lines: PC-3 (prostate cancer), MDA-MB-231 (breast cancer), A549 (lung cancer), IMR-90 (fetal lung fibroblast), and MCF-7 (breast cancer). Cells were infected with 1.0*10^7 ^IFU/mL and subjected to puromycin selection from 24 hours after infection. RNA was collected 48 hours (MCF-7), 72 hours (PC-3, MDA-MB-231 and A549) or 96 hours after infection (IMR-90). Relative mature miRNA expression was measured by qPCR. Values were standardized against the corresponding cell line infected with an empty vector control. We were able to overexpress all miRNAs in all cell lines, with the exception of miR-221 in IMR-90 cells (table 1). The fact that miR-221 is very effectively overexpressed in MCF7 indicates the expression construct is functional. Besides, we were able to overexpress all other miRNAs in IMR-90. The results with miR-221 can at least partially be ascribed to very high endogenous miR-221 levels in this cell line (additional file 2, table S2) and also suggest that overexpression of miRNAs using our standard conditions remains within physiological levels.

**Table 1:** Overexpression of miRNAs in various cell lines after lentiviral transduction

microRNA	cell line				
	PC-3	MDA-MB-231	A549	IMR-90	MCF7
miR-221	5.0	3.1	8.7	0.7	527
miR-203	> 1, 000	> 1, 000	> 1, 000	> 1, 000	45.6
miR-21	4.7	2.2	3.1	3.0	3.0
let-7a	11.8	114	18.9	17	38.3
miR-372	> 1, 000	> 1, 000	> 1, 000	> 1, 000	> 1, 000
miR-34a	> 1, 000	> 1, 000	26.0	17.6	170
miR-126	> 1, 000	> 1, 000	220	> 1, 000	293
miR-10b	> 1, 000	> 1, 000	321	406	> 1, 000
miR-335	> 1, 000	> 1, 000	> 1, 000	> 1, 000	> 1, 000
miR-205	> 1, 000	> 1, 000	14.1	> 1, 000	159

**Figure 3:**
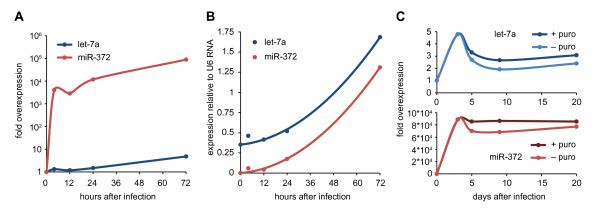
**miRNA overexpression kinetics**. A375 cells were infected with either let-7a or miR-372-encoding virus and expression was measured by miRNA qPCR. (A, B) Expression of let-7a and miR-372 is measured 0, 4, 12, 24 and 72 hours after infection. Data is plotted as fold overexpression (A) or relative to U6 snRNA expression (B). (C) Three days after infection, samples were passaged and cultured either with or without puromycin selection. Expression of let-7a and miR-372 was measured 5, 9 and 20 days after infection. For both miRNAs, expression dipped after three days but stabilized thereafter, resulting in a sustained overexpression.

Next, we examined miRNA expression kinetics after infection. To determine the optimal conditions for a screen, such as assay length and virus titer, it is important to know when and to what level the miRNA will be expressed. We have tested this extensively for let-7a and miR-372 in A375 cells. While let-7a is abundant in these cells, miR-372 is barely detectable (Figure 3). Both expression profiles show a quick surge of miRNA expression within 4 hours after infection. The early source of these miRNAs is unclear, but they may have been present in the virus supernatant, encapsidated within the virus, or processed intracellularly from the viral RNA genome. miRNA levels exhibit a slight drop, but are quickly overtaken by expression from integrated constructs 16 to 20 hours after infection. Our data demonstrate that already after 24 hours, ectopic expression of miRNAs reaches comparable levels of endogenously predominant miRNAs, as evident from the expression relative to endogenous let-7a and U6 RNA (Figure 3b).

For long-term experiments, it is necessary for miRNA expression to be sustained. 72 hours after infection with let-7a or miR-372, we passaged A375 cells and cultured them either with or without puromycin selection. We examined miRNA expression by miRNA qPCR at 5, 9 and 20 days after infection (Figure 3c). A decline in miRNA expression was observed with both miRNAs between day 3 and day 5 after infection. This may be caused either by passaging or changed culture conditions, but it is also possible that it is a secondary effect of highly elevated miRNA processing. This effect was markedly stronger in the let-7a-infected cells, which may indicate that there is a selection disadvantage for cells with high let-7a expression. Despite the small drop after three days, overexpression of both miRNAs stabilized and persisted for weeks. Even after prolonged culture without puromycin selection, miRNA overexpression was sustained. We conclude there is little or no genetic loss or epigenetic silencing of the integrated construct.

**Figure 4:**
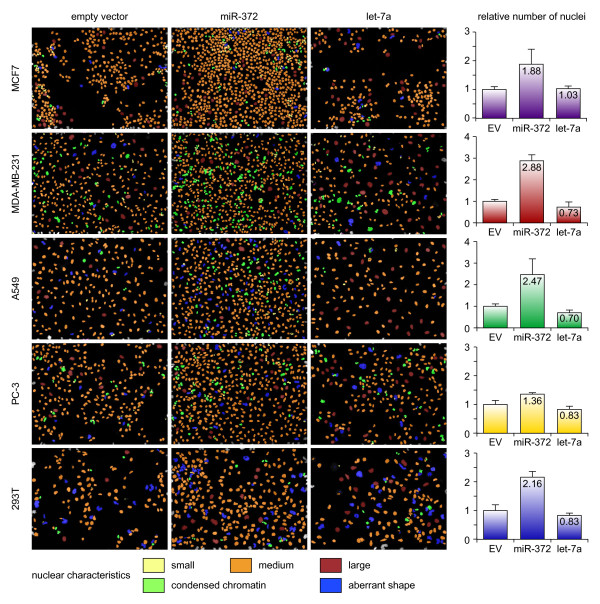
**Lentiviral transduction of miRNAs in a high-content setup**. Five different cell lines were infected with either empty vector miR-372, or let-7a. Cells were fixed 96 hours after infection and stained with Hoechst. Samples were analyzed with automated image analysis software. Four parameters were assessed: 1, number of nuclei per field; 2) nuclear size; 3) nuclear shape; 4) chromatin condensation. Graphs on the right show the quantification of the number of nuclei per field. Error bars represent standard deviations of triplicate wells. Four fields were counted per well.

As the goal of the library is to identify biologically relevant functions of miRNAs, we performed a proof-of-principle experiment by testing the well-documented effects of let-7a [49-51] as an inhibitor of cell proliferation and miR-372 [38] as a stimulator in five cell lines. The experiment was set up in the fashion of a high-content screen. Cells were seeded in 384-well plates and infected after 8 hours. 72 hours after infection, cells were fixed and nuclei were stained with Hoechst. The automated image analysis software not only counts the number of nuclei, but also detects aberrations in nuclear shape and condensed chromatin (Figure 4). Although we did not find any novel effects of the miRNAs on nuclear shape, we were able to confirm the expected effects of let-7a and miR-372 on proliferation in a high-content setup.

### Clonal screening

**Figure 5:**
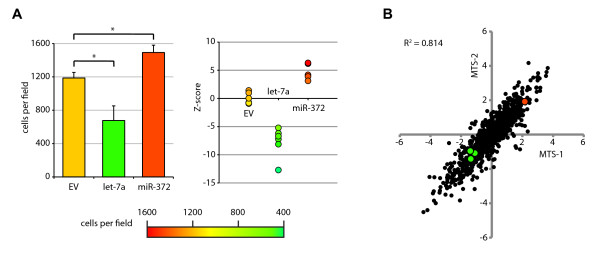
**Arrayed screening using the lentiviral library**. (A) A375 cells were seeded in a 96-well plate and infected with virus containing an empty vector, let-7a, or miR-372. Five days after infection, cells expressing let-7a were significantly less numerous than cells expressing empty vector, while cells expressing miR-372 were significantly more numerous (left panel, * indicates p < 10^-4 ^in a two-tailed t-test). Values are average number of cells per well ± standard deviation, n = 6. Each measurement of both let-7a and miR-372 samples is separated from the empty vector samples by more than three standard deviations (Z-score, right panel). (B) Results of an arrayed screen on cell viability of A375 cells. Cells in individual wells were infected with single miRNA constructs and subjected to an MTS assay 5 days after infection. The screen was performed in duplicate. For each measurement a B-score was calculated. Correlation between duplicate B-scores is shown. The green dots represent let-7a-1, -2, and -3, while the red dot represents miR-372.

As a first use of the complete library we screened miRNAs for their effect on A375 melanoma cell proliferation. We tested whether we could detect proliferation effects in A375 cells in a 96-well setup, again using let-7a and miR-372 as test miRNAs. The effects were highly similar to those seen in Figure 4 (Figure 5a). Every single well infected with either let-7a or miR-372 proved a statistical outlier compared to empty-vector transduced wells (Figure 5a, right panel).

For the entire library, we performed an arrayed screen by MTS assay, which measures the viability of cells in a well [52]. This measure is reported to correlate strongly with cell number. All samples were tested in duplicate, and for each replicate a B-score was calculated [53]. This method calculates how a miRNA scores relative to the other miRNAs on the same plate. It is a robust method as it is not sensitive to outliers. B-scores of replicates are shown in Figure 5b. Also shown are the measures for all let-7a constructs and miR-372. The strong correlation between replicates indicates a high reproducibility of the assay. The effects of let-7a and miR-372 are similar as in the cell count assay. Importantly, all let-7a constructs cluster together. Since these constructs have different backbones but produce the same mature miRNA, we surmise the observed effects are likely to be caused by the mature miRNA. A confounding factor in short-term arrayed screens can be the range of virus titers of the different constructs. However, we saw no correlation between virus titer and cell viability (additional file 3, figure S1). Thus, with these experimental conditions, virus titers were not a significant factor contributing to toxicity.

### Pooled screening

**Figure 6:**
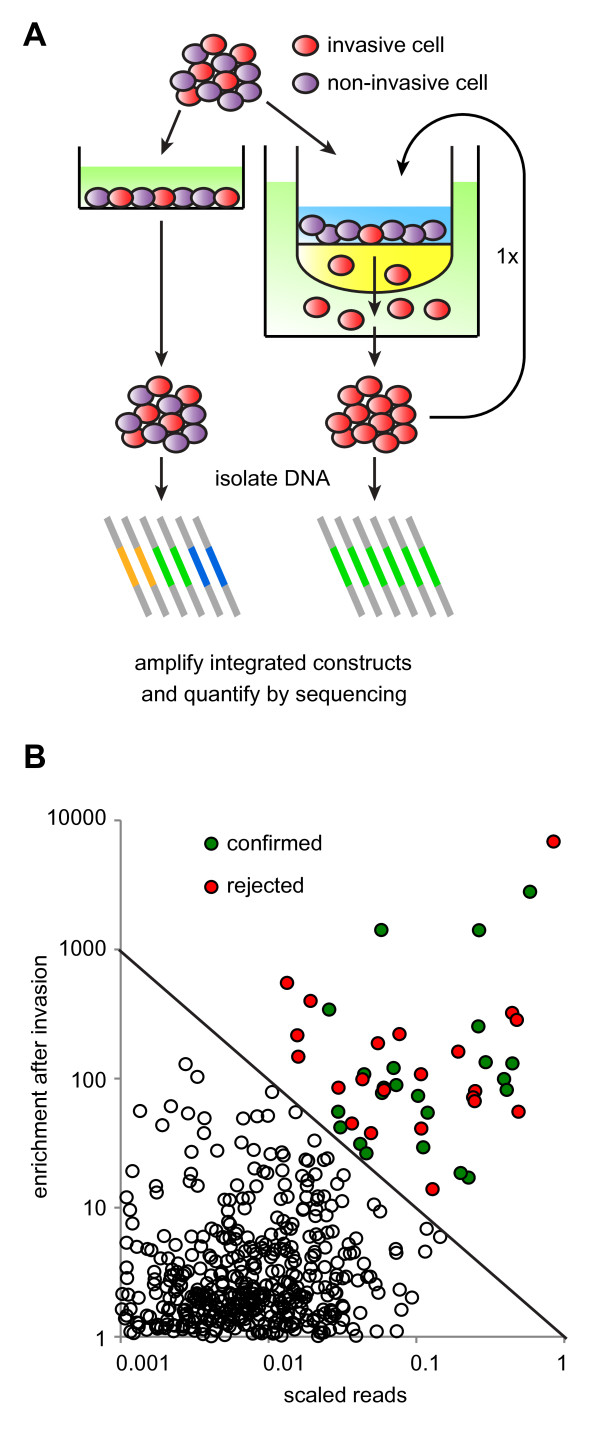
**A pooled screen for invasive capacity of miRNAs using the lentiviral library**. (A) Schematic of the pooled invasion screen setup. In brief, after clonal infection (not shown here), cells with 40 individual miRNA constructs were pooled and either subjected to two rounds of an invasion assay or used as the control fraction. Of both fractions, integrated constructs were quantified by massively parallel sequencing to determine enrichment. (B) miRNAs were chosen for confirmation based on a combination (E × R > 1) of enrichment (E) after invasion and their abundance in the invasive fraction; expressed as the scaled number of reads (R). 23 out of 45 miRNAs were confirmed to enhance invasive capacity of HCT15 cells in final individual invasion assays.

Some assays do not lend themselves to screening in 96-well format. In such cases the lentiviral library can be used for a pooled screen. There are two approaches: pool virus supernatant of several or all constructs together, or infect cells with the individual constructs and subsequently pool cells. The latter is generally favorable, because it precludes the possibility that two or more virus particles containing different miRNAs infect a single cell. A puromycin selection step can be added to make certain each construct is represented in transduced cells and to reduce background caused by non-transduced cells. We applied this approach to the poorly invasive HCT15 colorectal cell line. HCT15 cells were individually infected, puromycin-selected, and pooled in groups of 40 constructs. A fraction of the pool was used for genomic DNA isolation and the rest was subjected to two rounds of an invasion assay. Cells that successfully transmigrated through extracellular matrix were subcultured and genomic DNA was isolated. To identify the miRNA constructs that were enriched in the transmigrated fraction, we took advantage of the fact that the constructs are integrated into the host genome. We PCR-amplified the constructs from the genomic DNA using universal primers that anneal to the vector backbone (Figure 6a). The PCR products were subjected to high-throughput sequencing. Relative abundance of the various constructs was derived from the sequence reads mapping to them. Hits were selected on the basis of a weighted score calculated from the enrichment and the number of sequence reads in the invasive fraction. We found evidence for 45 miRNAs that may enhance the invasive capacity of HCT15 cells (Figure 6b).

In a pooled invasion screen, as performed above, the possibility exists that a portion of the hits can be "passenger hits" that do not impart invasive capacity, but are present in cells that trail the truly invasive cells as they make their way through the extracellular matrix. To distinguish these two types, we subjected each hit individually to an invasion assay. We were able to confirm 23 miRNAs that increase invasiveness of HCT15 cells (Figure 6b).

## Conclusions

We present a lentiviral miRNA expression library that is optimized for use in arrayed screens. The library allows miRNAs to be individually assessed in any gain-of-function screen. We envision the library being used in a wide range of functional screens. To name some possibilities: reporter-based assay screens, screens to study cell cycle, senescence, and epithelial-to-mesenchymal transition. The broad tropism of the lentivirus makes it applicable to infect cells of different species. This may prove particularly valuable, since lentivirus can be directly used *in vivo *[43]. Thus, the same lentivirus can be used to screen for a miRNA function *in vitro *and verify this functionality *in vivo*.

We have demonstrated the transduction of stem cells that were subsequently used for *in vitro *organ culture. The ability to stably transduce stem cells opens up avenues for studying miRNAs required for differentiation and stemness.

An arrayed library comes with several advantages. Arrayed screening offers more sensitivity and faster results than pooled screening, without the need of data deconvolution. It also allows for the assessment of several more complex parameters, as is commonplace in high-content screens. Assays that require long-term culture may not be amenable to arrayed screening and require pooling. Still, infecting cells individually before pooling is likely to decrease false-negative results in pooled screens, suggesting that even for pooled screens an arrayed library may be preferred.

Our library currently contains the majority of all human miRNAs. However, the most recent update (version 17) of miRBase includes a large number of newly identified miRNAs. While we do not wish to question their validity or understate their potential, we argue that most miRNA functions will be covered by the most highly expressed or broadly conserved miRNAs, which were already present in earlier versions of miRBase and thus represented in our library. By the same token, the merit of candidate miRNAs in our library can be questioned. In anticipation of this, candidate miRNAs were positioned on separate plates in the library, presenting researchers the choice to screen the entire library, only the annotated miRNAs, or only the candidate miRNAs.

Expression of the miRNAs from their native genomic background ensures the physiological processing of the miRNA. Not only the pre-miRNA hairpin, but also the flanking sequence contributes to proper processing [54]. Transcribing the miRNA from an integrated construct enables expression of both arms and all isomiRs that would naturally derive from the primary transcript. Therefore, the library could be employed for further characterization and validation of the included miRNAs. Such experiments have been extensively done for mouse miRNAs [55], but not for human miRNAs.

We have shown that ectopic expression of miRNAs using a lentiviral vector can be used to screen for biologically relevant effects. While the library is widely applicable and can be used to study various aspects of biology, our primary focus is on cancer-related processes. A first screen assessing miRNA-induced effects on melanoma cell growth demonstrated the value of the library in an arrayed screen. The next step is to perform such screens over a panel of cell lines to determine which miRNAs may have a growth effect on specific cancer indications, and which miRNAs have a general growth inhibitory or stimulatory function. These results will aid in finding miRNAs suited for tumor-specific treatment. Indeed, such therapeutic options have been successfully explored in a murine model of hepatocellular carcinoma [56]. With the miRNA expression library, we offer a platform that facilitates the identification of miRNAs with therapeutically relevant functions.

## Methods

### Construction of the lentiviral library and other constructs

Backbone for all constructs in the library is the lentiviral expression construct pCDH (cat. no. CD510B-1, System Biosciences). Individual loci containing a single miRNA hairpin were PCR amplified from human genomic DNA and cloned into the multiple cloning site of the plasmid. Loci were either obtained from miRBase http://mirbase.org/ or from mapping of candidate miRNAs found in previous experiments [10,40]. Primers were designed using Primer3. PCR was performed using Pfu polymerase (Agilent). The cloned fragments contain the full-length miRNA hairpin and approximately 100 flanking base pairs on both sides. An expression construct with EGFP was cloned by excision and ligation of the EGFP sequence from pEGFP-N1 (Clontech) into the multiple cloning site of pCDH. All constructs were packaged into lentiviral particles commercially by System Biosciences using the pPACKH1 HIV-based lentiviral packaging kit (cat. no. LV500A-1, System Biosciences). Viral particles were recieved in concentrated form with a median titer of 5.9*10^8 ^IFU/mL in a 96-well format. Sequence of all inserts was confirmed from both the plasmid and the virus supernatant (we were able to obtain high-quality specific sequences from the virus supernatant using universal primers, most likely due to trace amounts of plasmid). A list of all included miRNAs in their current annotation (miRBase17) is available in additional file 1, table S1. Lentiviral particles for pCDH with copGFP instead of puroR (cat. no. CD511B-1) were ordered separately from System Biosciences.

### Cell culture and viral infections

293T, A375, MDA-MB-231, A549, MCF-7, IMR-90, PC-3, and HCT15 cells were maintained on 10% FCS complete medium: DMEM Glutamax (GIBCO) with 10% FCS (Sigma) supplemented with non-essential amino acids (GIBCO) and penicillin/streptomycin (GIBCO). All viral infections followed by RNA isolation were done 8 hours after seeding 10, 000 cells in a 6-well plate in 2 mL 10% FCS complete medium. All transduction efficiency experiments were performed by infection with pCDH-MCS-EF1-copGFP, except for the organoid culture, for which we used pCDH-EGFP-EF1-PuroR. Infection mix contained 2 μL virus supernatant, 12 μL 1 mg/mL polybrene (Sigma), and 86 μL PBS0, unless stated otherwise. Intestinal organoid bodies were cultured as described before [48]. Because the organoids are grown in Matrigel, cells were infected before seeding. Cells were infected in 250 μL Wnt-3a-conditioned medium containing 2 μL virus supernatant. Infection took place during a centrifugation step at 150 rcf for 1 hour at room temperature. Unless indicated otherwise, infections were performed in 96-well plates using the following set-up: 1000 cells were seeded in 100 μL 5% FCS complete medium per well of a 96-well plate and infected after 8 hours with 10 μL infection mix. 10 μL infection mix contained 0.6 μL 1 mg/mL polybrene, 0.5 μL virus supernatant, and 8.9 μL PBS0. In the arrayed screen, medium was replaced with 150 μL fresh medium 24 hours after infection.

### MTS assay

Five days after infection, 30 μL MTS One Solution (Promega) was added to all samples. After each hour, plates were gently tapped to disperse the coloration of the MTS, and absorbance was measured at 492 nm. Last measurements were taken 4 hours after start of the assay. The time point with measurements showing the highest dynamic range without saturation of signal was used for data analysis.

### Nuclear staining, GFP quantification and high-content applications

Five days after infection, 100 μL 8% PFA (Sigma) was added to all samples. Cells were fixed for 15 minutes. Samples were washed once with PBS0 followed by 10 minutes staining in 100 μL PBS0 containing 0.5 μg/mL Hoechst 33342 (Sigma). Cells were washed twice with PBS0 and kept on PBS0 at 4°C. Cells were quantified on a Cellomics ArrayScan VTI using the accompanying software by counting nuclei in 4 fields per well under 10× magnification. Nuclei were identified as shapes with a contiguous Hoechst stain. Nuclear demarcations were used to quantify GFP intensity. All data acquisition was done using adaptions of the TargetActivation program of the ArrayScan software. Data and images displayed in table 1 and Figure 4 were generated by Cenix BioScience, GmbH.

### RNA isolation and miRNA qPCRs

RNA was isolated using TRIzol (Invitrogen) isolation following the manufacturer's protocol. Small RNAs qPCR reactions were performed using the TaqMan MicroRNA reverse transcription kit (ABI), Taqman MicroRNA qPCR assays (ABI) and TaqMan Universal PCR Master Mix, No AmpErase UNG (ABI) using 10 ng total RNA input. qPCR reactions were set up using the suggested reaction conditions on a Bio-Rad MyiQ thermal cycler. U6 was used as a housekeeping control RNA in the experiments concerning Figure 3, RNU6B was used in the experiments concerning table 1. Relative expression was calculated using the 2^-ΔΔCt ^method. miRNA qPCR data in table 1 were generated by Cenix BioScience GmbH.

### Invasion assay

HCT15 cells were seeded at 2500 cells per well in a 96-well plate in 10% complete medium. Cells were infected with individual virus supernatants (0.5 μL per well) after 24 hours. 72 hours after infection cells were selected with puromycin-containing medium and subsequently grown to 100% confluency. Pools were made of 40 samples per pool and grown for another 3 days on puromycin-containing medium. Half of the pooled culture was used for genomic DNA isolation, the other half was subjected to two rounds of invasion assay. For each pool, 2*10^5 ^cells were applied to the upper compartment of a Boyden chamber (BD Fluoroblok 24-Multiwell, 8 μm pores) coated with extracellular matrix (ECM, Sigma) and containing serum-free medium, and allowed to invade the lower compartment containing 10% FCS medium. Cells were collected from the bottom compartment and expanded for 2-3 weeks on complete medium. 2*10^5 ^cells of this subculture were applied to a second round of invasion as described above. Cells in the resulting bottom compartment were subcultured to be used for genomic DNA isolation.

### Genomic DNA isolation and massively parallel sequencing

Genomic DNA was isolated from cells using a Qiagen Genomic Tip kit following the manufacterer's instructions. For both the invasive fraction and the control fraction, 10 ng DNA of each pool was pooled together and subjected to PCR-amplification (20 cycles) of integrated constructs using Platinum PCR Supermix (Invitrogen) and primers flanking the inserts. In a subsequent, secondary (3 cycles) and tertiary (3 cycles) PCR the adapters and barcodes used for massively parallel sequencing were added to the flanks of the products. Samples were sequenced on the ABI SOLiD platform. The invasive and control fraction yielded 1.0*10^6 ^and 0.9*10^6 ^reads that mapped to integrated constructs respectively.

### Library availability

Both the plasmid and virus library are publicly available when requested through InteRNA Technologies. Contact information is available at http://www.interna-technologies.com.

## Authors' contributions

JBP constructed the plasmid library, designed and performed most experiments, analyzed all data and drafted the manuscript. RJvH constructed the plasmid library and designed and performed the arrayed screen. FC, ASB, LMT, BD and GAM designed and/or performed the pooled screen and its confirmation follow-up. AAFLvP was involved in the design of the library validation experiments and the arrayed screen. EB, RQJS and EC conceived and designed the lentiviral library. EC supervised all studies and critically revised the manuscript. All authors read and approved the final manuscript.

## Supplementary Material

Additional file 1 **Table S1**. Contains a list of the miRNAs included in the library and their cognate genomic sequences that were cloned into the vector, and associated virus titers. Click here for file

Additional file 2 **Table S2**. Contains the endogenous levels of the set of 10 test miRNAs in the five tested cell lines. Expression calculated as 2^-ΔCt ^relative to RNU6B. Click here for file

Additional file 3 **Figure S1**. Demonstrates the lack of correlation between virus titer and toxicity. Click here for file
