# Supplementary material for: Functional microRNA screening using a comprehensive lentiviral human microRNA expression library
Source: BMC Genomics. 2011 Nov 3;12:546. doi: 10.1186/1471-2164-12-546 (PMC3227672; doi:10.1186/1471-2164-12-546)
Supplement: Additional file 3 — Figure S1. Demonstrates the lack of correlation between virus titer and toxicity. [file 1471-2164-12-546-S3.PDF]

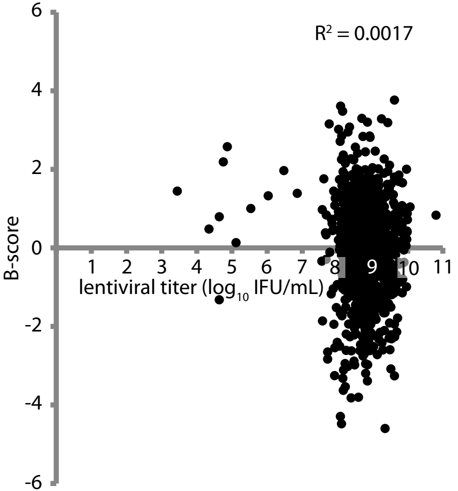

Supplementary figure 1. Virus titer and cell viability show no correlation. Averaged B-scores from the arrayed screen were plotted against the virus titers of the corresponding constructs. We observed no correlation between virus titer and cell viability, indicating that virus titer does not contribute significantly to toxicity in this screen.
